# Prevalence of Aflatoxins in Camel Milk from the Arabian Peninsula and North Africa: A Reduction Approach Using Probiotic Strains

**DOI:** 10.3390/foods12081666

**Published:** 2023-04-17

**Authors:** Tawfiq Alsulami, Mohamed G. Shehata, Hatem S. Ali, Abdulhakeem A. Alzahrani, Mohamed A. Fadol, Ahmed Noah Badr

**Affiliations:** 1 Food Science & Nutrition Department, College of Food and Agricultural Sciences, King Saud University, Riyadh 11451, Saudi Arabia; 2 Food Technology Department, Arid Lands Cultivation Research Institute, City of Scientific Research and Technological Applications(SRTA-City), Borg El Arab 21934, Egypt; gamalsng@gmail.com; 3 Food Research Section, R&D Division, Abu Dhabi Agriculture and Food Safety Authority (ADAFSA), Abu Dhabi P.O. Box 52150, United Arab Emirates; 4 Food Technology Department, National Research Centre, Dokki, Cairo 12622, Egypt; hatem.owyean1@gmail.com; 5 Food Toxicology and Contaminants Department, National Research Centre, Dokki, Cairo 12622, Egypt

**Keywords:** aflatoxin M_1_, aflatoxin B_1_, aflatoxin removal, antioxidant activity, camel milk, ELISA technique, feed contamination, probiotic bacteria

## Abstract

Camel milk is known as a source of nutritional and health supplements. It is known to be rich in peptides and functional proteins. One main issue facing it is related to its contamination, mainly with aflatoxins. The present study aimed to evaluate camel milk samples from different regions while trying to reduce its toxicity using safe approaches based on probiotic bacteria. Collected samples of camel milk were sourced from two main regions: the Arabic peninsula and North Africa. Samples were tested for their contents of aflatoxins (B_1_ and M_1_) using two techniques to ensure desired contamination levels. Additionally, feed materials used in camel foods were evaluated. Applied techniques were also tested for their validation. The antioxidant activity of camel milk samples was determined through total phenolic content and antioxidant activity assays. Two strains of probiotic bacteria (*Lactobacillus acidophilus* NRC06 and *Lactobacillus plantarum* NRC21) were investigated for their activity against toxigenic fungi. The result revealed high contamination of aflatoxin M_1_ for all samples investigated. Furthermore, cross-contamination with aflatoxin B_1_ was recorded. Investigated bacteria were recorded according to their significant inhibition zones against fungal growth (11 to 40 mm). The antagonistic impacts were between 40% and 70% against toxigenic fungi. Anti-aflatoxigenic properties of bacterial strains in liquid media were recorded according to mycelia inhibition levels between 41 to 52.83% against *Aspergillus parasiticus* ITEM11 with an ability to reduce aflatoxin production between 84.39% ± 2.59 and 90.4% ± 1.32 from media. Bacteria removed aflatoxins from the spiked camel milk in cases involving individual toxin contamination.

## 1. Introduction

Milk is a nutrient-rich beverage that possesses health benefits. Milk contains essential nutrients, minerals, and vitamins and is also considered an excellent source of protein. Generally, it is recognized as a nutrient-rich fluid produced by female mammals to feed their offspring. The most commonly consumed types of milk are buffalo, sheep, goat, and cow’s milk, with cow’s milk being favorable in Western countries [1]. Dairy consumption is sometimes a controversial issue, raising the critical question of whether milk consumption is healthy or a source of harm. Camel milk forms the dietary habits of global nomads and desert populations with all the nutrients represented in other milk varieties [2]. Both fresh and fermented, camel milk has been consumed for human nutrition and for illnesses treated in traditional medicine [3]. Evidence suggests that camel milk has immunomodulatory effects and is readily absorbed by the body. Children who lack the enzyme lactase and are allergic to cow’s milk do well on a diet of camel milk. There is evidence that drinking camel milk may help protect the body from the harmful effects of toxins and microbial infection [4].

Like other types of milk, camel milk is a metabolite secreted by the mammalian gland, which is affected by feed ingredients and any potential contamination. Feed contaminants that can pass into the excreted milk include heavy metals, pesticide residues, hormones, and mycotoxins. These contaminants indirectly threaten public health due to their accumulation in small quantities via regular consumption of milk and dairy products. Mycotoxins represent the most significant danger among these contaminants due to their classification by the International Agency for Cancer Research as pre-carcinogens. Mycotoxins are classified into 400 types, of which the most serious to public health are aflatoxins (AFs). Due to cross-contamination, milk can be infected with aflatoxin (B or G) types. It also may be contaminated with metabolic products from feed contamination, as in aflatoxin M types.

Recently, probiotics and lactic acid bacteria have been used as influential factors in reducing contamination in dairy products [5]. Previous studies indicated the role played by these strains due to their activity through antifungal action or their role in reducing the secretion of mycotoxins [6,7]. Two probiotic strains were recorded with antifungal activity via a simulated in vivo investigation [8]. The previous investigation reveals the functionality of some strains of probiotic bacteria (*lactobacillus acidophilus* and *bifidobacteria*) in the reduction of AFM_1_ contamination [9]. It should be noted that three different mechanisms can explain the in vivo action of bacterial strains against mycotoxins. Bacterial cell walls can chelate mycotoxins and generate a complex that facilitates removal throughout the biological system [10]. In this method, mycotoxins can leave the body securely, preventing them from causing tissue injury. Other mechanisms are linked to bacterial metabolites [11].

Camel milk is consumed in the middle East and Arab regions in considerable quantities. It is handled in markets and sold for local consumption in some areas such as Saudi Arabia. A few investigations discuss this point, but none recommend a solution. The study investigated aflatoxin contamination in camel milk, which is known to be used for nutritional and immunological consumption. The research was targeted to explore the current situation of aflatoxin (AF) contamination in commercial samples of camel milk. Also attempted to find the link between the source of feeding and the contamination levels and reduce these contamination levels using a safe approach. Additionally, provides solutions to consuming camel milk with a low hazard of mycotoxin contamination. The fermentation using two probiotic strains was applied as a part of the strategy to enhance product safety. 

## 2. Materials and Methods

### 2.1. Sample Collection

Camel milk samples were collected as commercial samples from markets in the Arabian Peninsula (Saudi Arabia and United Arab Emirates) and North Africa (Egypt and Libya). Samples were collected in 1 kg quantities each (5 samples/region), and each country was classified into three areas.

Using the same manner described above, we also collected samples of feed materials to evaluate contamination levels of camel milk. Two feed materials were collected: ready-to-use (imported manufactured) feed and wild, green-feed materials. The aflatoxin content of evaluated feed materials was utilized to further recommend healthy camel milk consumption.

### 2.2. Materials and Chemicals

Microbiological media, including potato dextrose agar (PDA), De-man Rogosa and Sharpe (MRS), yeast extract agar (YES), potato dextrose broth (PDB), and Czapek-Dox agar (CDA) were BD Difco analytical media acquired from Fisher Scientific, Guldensporenpark, Merelbeke, Belgium. Methanol, Ethanol, Di-methyl-sulfoxide (DMSO), Trolox (6-hydroxy-2,5,7,8-tetramethyl chroman-2-carboxylic acid), ABTS+ (2,2′-azino-bis-3-thylbensothiazoline-6-sulfonic acid), DPPH (2,2-diphenyl-1-picryl-hydrazine-hydrate), and other solvents applied were of analytical grade, Merck Co., Ltd., Burlington, MA 1803, USA.

Two ELISA kits (an aflatoxin M_1_ Kit and a total aflatoxin kit) were applied to determine AF content. The provided materials inside the Elabscience test kit^®^ (14780 Memorial Drive, Houston, TX 77079, USA) included a Microtiter plate pre-coated with linked antigen, Horseradish peroxidase conjugate (HRP), AF standard solutions required to generate a calibration curve, chromogen (tetra-methyl-benzidine), and a stop reagent.

### 2.3. Sample Preparation for the Analysis

Before the AF determination, collected samples were prepared according to the methodology described by the manual of the applied technique of the ELISA kits. The milk sample was centrifuged (5000× *g*/10 min/4 °C) for the cream separation, the formed cream layer was discarded, and 40 µL of milk was taken for analysis using the ELISA technique. Feed samples (1 g) were ground with aqueous methanol (10 mL; 80%) and 0.1 g of NaCl. The slurry was filtered using filter paper (Whatman no.1), followed by the 0.45 µm filter, where the filtrate cleanup was completed using an AflaTest^®^ column. The column was washed twice before aflatoxin was eluted with 1 mL methanol (HPLC grade). A quantity of 40 µL was applied in the same way as it was for the milk-analysis step.

### 2.4. Determination of Aflatoxin Content Using the ELISA Technique

The aflatoxin content for the collected samples was determined according to the methodology described in the kits’ manuals. The samples and standard solutions were injected into prepared plate wells. A total of 80 µL of the HRP solution was added to wells that were immediately sealed and oscillated (10 s) before undergoing shaded incubation (40 min). When the incubation ended, wells were washed using 260 µL of washing buffer (4 replicates, intervals of 30 s) and inverted for the drying step. Reagent A (50 µL) and reagent B (50 µL) were added to each well, and the plate was re-incubated (15 min; 25 °C) for the reaction performance. The stop reaction solution (50 µL) was added to each well when the reaction time ended, and the optical density was immediately measured for the wells at 450 nm. A calibration curve was performed using the standard concentration of kits to calculate aflatoxin concentrations.

### 2.5. Determination of Aflatoxins Using the VICAM Technique

The aflatoxin content was determined following the methodology described previously [12]. In summary, 100 g (100 mL) of representative samples was blended with 10 g of NaCl and 200 mL of 80% aqueous methanol. The slurry was homogenized for one minute using a high-speed blender and then filtered using Whatman paper (No. 1). Before re-filtering, the filtrate (5 mL) was diluted with Milli-Q water (20 mL). Ten milliliters of the filtrate was purified on a VICAM immunoaffinity column (Aflatest, VICAM, Milford, MA, USA). The column was washed with 10 mL of Milli-Q water before the aflatoxin was eluted with 1 mL of methanol. The eluted fraction was measured with the VICAM fluorometer after diluting twice with Milli-Q water (VICAM Series 4EX Fluorometer). All operations were carried out following the manufacturer’s instructions.

### 2.6. Validation of the Applied Methodologies

Before analyzing the samples, the ELISA and VICAM techniques were tested to guarantee the validity of the findings. Validation of ELISA was accomplished by calculating recoveries. The mean coefficient of variation for fresh milk spiked with varying concentrations of AFs (10, 20, 40, 80, and 160 ng/L), and results are summarized below in Section 3 of the results.

### 2.7. Determination of Antioxidant Activity in Camel Milk

Total phenolic content, DPPH (2, 2-diphenyl-1-picryl-hydrazyl-hydrate free radical method), and ABTS+ scavenging (2, 2′-Azinobis [3-ethylbenzothiazoline-6-sulfonic acid]-diammonium salt) were determined to indicate camel milk’s antioxidant activity. The previous methodology (with modifications) was followed to evaluate the antioxidant activity of camel milk [13]. Collected samples were first centrifuged (5000× *g*/4 °C/10 min) to separate the cream layer. Briefly, phenolic content was measured in milk before and after bacterial fermentation. Creamless milk (1 mL) was blended with ethanol (1.0 mL, 95% *v*/*v*) and deionized water (5 mL). The Folin–Ciocalteu reagent (0.5 mL; 50% *v*/*v*) was added to each sample, and after vigorous mixing, the solutions were let to stand (25 °C/5 min). Sodium carbonate solution (1.0 mL, 5% g/100 mL) was added, and then the absorbance was measured after an hour of incubation (at 725 nm). The total phenolic content was measured as a microgram equivalent of Gallic acid (μg GAE/mL milk).

DPPH inhibition was determined by mixing 250 μL of milk with DPPH (3 mL of 60 mmol/L in ethanol) [14]. The mixture was shaken thoroughly and stood (25 °C/20 min). The absorbance readings were measured (at 517 nm), and the DPPH inhibition (%) was calculated as follows:

(1) %DPPH=(Ac−As/Ac)×100,

where Ac represents absorbance of the control, and

As represents absorbance of the sample.

The same manner was applied for the ABTS+ scavenging determination with the required suitable solutions described previously [15], and the absorbance was measured at 734 nm. The inhibition was expressed according to the following equation:

(2) %ABTS+=(Ac−As/Ac)×100

where Ac represents absorbance of the control, and

As represents absorbance of a sample.

### 2.8. Activation of Bacterial Strains

Two strains of probiotic bacteria, *Lactobacillus acidophilus* NRC 06 and *Lactobacillus plantarum* NRC 21, were gifted from the Dairy Department, National Research Centre, Cairo, Egypt. The strains were reactivated once in sterile skimmed milk media (11%) and twice in MRS media before the application. The bacterial concentration was adjusted using a hemocytometer chamber at 1.3–1.7 × 10^9^ CFU/mL.

### 2.9. Preparation of Bacterial Supernatant

Bacterial supernatant was prepared with the bacterial growth in 1 L of the MRS media [16]; the bioactive components were regained using a dichloromethane and media broth mixture at a ratio of 3:1. The supernatant was recovered using a rotary evaporator (Heidolph, HeiVAP, GmbH, Landsberger, Germany). It was kept in an amber vial until further applications.

### 2.10. Determination of the Antifungal Effect

The antifungal effect of bacterial strains and their supernatants was evaluated against fungal strains of toxigenic fungi [17]; these strains are known to contaminate camel feed material. The toxigenic fungal strains were *Aspergillus flavus, A. parasiticus, A. niger, A. fumigatus, Penicillium oxysporium, P. notatum, Fusarium graminaerum*, and *Alternaria alternate.* These strains were isolated from feed materials and identified by the Food Toxicology and Contaminant Department, NRC, Egypt.

The ability of bacterial strains to suppress isolated toxigenic fungal growth was investigated [18]. The bacterial antagonism was performed in vitro using PDA media on Petri dishes. On a Petri plate, a disc of fungi was inoculated on one side, whereas a bacterial disc was inoculated on the other. Suitable distances were left between each bacterial culture site and the Agar discs of the examined fungus. Negative control of fungal agar discs without bacterial culture spots was performed. The Petri plates were then incubated (5 days/30 °C). The percentage of fungal growth inhibition was estimated using the following formula:

(3) $$\%A=\left(1-\left(\frac{X}{Y}\right)\right)\times 100$$

where %A: represents antagonism ratio,

X: represents the distance between the fungal edge and bacterial edge, and

Y: represents the distance between the treated fungi’s upper edge and the control’s upper edge.

The well-diffusion assay was applied to evaluate the antifungal activity of the bacterial supernatant; each well was loaded with 100 µL of bacterial supernatant, as described previously [19]. The results were expressed as millimeter diameters (mm) of the inhibition zone achieved around the well; the greater the inhibition diameter, the more efficient the supernatant.

### 2.11. Determination of the Anti-Aflatoxigenic Effects

The anti-aflatoxigenic effects of bacterial strains were evaluated using the YES media containing a productive fungal strain of *A. parasiticus* ITEM 11, as described previously [20]. The experiment was divided into two major groups of flasks. The first group used the fungal growth in the presence of a bacterial strain using potato dextrose broth (PDB) media to suit the two microorganisms. This group comprised two flasks infected with fungus (1.37 × 10^3^ CFU/mL) and bacteria (1.71 × 10^9^ CFU/mL), whereas the control flask was inoculated with fungi. Flasks were incubated (30 °C/5 days), and mycelial reduction was expressed as dry weight and a ratio of inhibition against the control.

The second group was tested after the fungus was grown and mycelia were discarded. *A. parasiticus* fungal spores inoculated the flasks containing YES broth. The flasks were incubated (30 °C/9 days) to enable aflatoxin production [19]. By the end of incubation, the media were filtered using Whatman (No. 1) filter paper followed by a micro syringe filter (Millipore, 45 µm). Bacterial strains were enriched on MRS media (24 h) and centrifuged to collect the bacterial pellets that inoculated to the filtrate of fungal media. The flasks were incubated (37 °C/2 h) before measurements of the aflatoxin content were taken. Aflatoxin concentrations in media were measured before and after bacterial pellet treatment.

### 2.12. Application of Bacteria for Milk Fermentation

Bacterial strains of *Lactobacillus acidophilus* NRC 06 and *Lactobacillus plantarum* NRC 21 were utilized in camel-milk fermentation. Samples of camel milk were spiked with aflatoxin M_1_ (220 ng/mL) and Aflatoxin B_1_ (400 ng/mL). Camel milk was packed in sterile bottles, inoculated with bacteria at 1.7 × 10^9^ CFU/mL, incubated (37 °C/2 h), and then cooled overnight. Camel milk was inoculated with bacteria strains as individuals and as a mix. Aflatoxin concentrations were measured in samples after 24 h of treatment.

### 2.13. Statistical Analysis

At least three results were given as means ± standard deviation (SD). ANOVA was used to determine if there was a significant difference between the means, and Duncan’s multiple range test (*p* = 0.05) was performed. The SPSS V.22.0 and Graph Pad Prism V.7.0 statistical programs were used to analyze the data expressed as means ± SD.

## 3. Results

Collected samples from the four countries were inspected concerning the presence of AFs for knowledge of potential contamination in camel milk. The results also illustrate the variation in aflatoxin content in camel milk of the Arabian Peninsula and North African regions. Moreover, two feed material sources, including dry imported and wild plant feeds, were analyzed to detect potential contamination hazards. To our knowledge, wild plants are the primary feed material consumed in North Africa, and imported dry feed is the primary feed material consumed in the Arabian Peninsula.

### 3.1. Aflatoxin Determination

#### 3.1.1. AFM_1_ Evaluation in Camel Milk

The AF content of camel milk was determined to identify natural contamination caused by the AFM_1_ toxin and to check for the occurrence of cross-contamination with the AFB_1_ toxin. Table 1 shows the AF contamination for the collected samples determined using two techniques (ELISA and VICAM). A high presence of aflatoxin contamination was demonstrated in collected samples from the coastal region (Region 1). The farthest area of the coast seemed to have the lowest contamination level (Region 3). For the samples collected in the United Arab Emirates, there were no significant differences between the region samples concerning aflatoxin _M1_ content. This result could be explained by the fact that these samples were taken from the most extended coastal areas occupying a narrow geographical region.

The AFM_1_ contamination levels recorded in the United Arab Emirates and Egypt samples seemed similar in Region 1. We noticed that the primary type of feed in these areas was dried-manufactured feed without any natural feed from wild plants [21]. Camel milk samples from Region 3 in Egypt, which mainly utilized wild plants in camel feeding with little dry-feed material, recorded lower AFM_1_ contamination. Bedouin pastoralists in these areas referred to their dried feed as partial consumption due to the dried climate seasons and rarely found wild plants. In Libya, wild pastoralism was found to be the primary type. This behavior may explain the lowest contamination levels of the AFM_1_ in camel milk samples from this area.

#### 3.1.2. AFB_1_ Evaluation in Camel Milk

The main cause of the AFM_1_ contamination was AFB_1_ as it transformed metabolically from contaminated feed consumed by the mammalian, resulting in AFM entering the animals’ bodily fluids. Furthermore, AFB_1_ could have been present through cross-contamination in milk samples during handling, transportation, or storage. Collected samples were inspected for AFB_1_ cross-contamination, and the results reflect its occurrence in all camel milk samples (Table 2).

The cross-contamination levels with the AFB_1_ in the investigated camel milk samples were similar. This result reflects the need to pay attention to hygiene practices during the production and product-handling stages. The contamination levels were remarkable and exceeded the permissible limits in the collected samples. This indicates the need to review the stages of production and storage well to preserve the therapeutic properties of this type of milk. It is known that camel milk is healthy and can be relied upon to boost immunity levels as it is rich in vital peptides and functional proteins. However, the accidental or direct contamination of these kinds of milk may make it a source of hazard to public health. The risk of this contamination is related to mycotoxins as they are invisible and require specialized approaches for detection. Therefore, the best practice is to check and adequately review the stages of production and the quality of feeding to reduce contamination incidence caused by mycotoxins.

### 3.2. Method Validation of Aflatoxin Determination

The validity of the method was first evaluated using spiked aflatoxin concentrations for aflatoxin M_1_ (AFM_1_) and aflatoxin B_1_ (AFB_1_). The determination results are recorded in Table 3, wherein the recovery seems acceptable for accurately evaluating aflatoxin content.

It was noticed that the recovery at different concentrations showed acceptable levels, and few changes were recorded regarding the factor influencing coefficient variation. The results at this stage provide clarity regarding the aflatoxin evaluation.

### 3.3. Aflatoxin Determination in Plant Feeds

Feed samples were investigated for sources of risks that may be linked to AFM_1_ in camel milk. First, wild plant samples consumed in natural pastoralism contexts were analyzed, and the results are shown in Table 4. AFB_1_ was present in collected plant material during the investigation; however, AFB_1_ was present in samples at low levels. Determination of the changes in AFB_1_ using the two techniques of ELISA or VICAM revealed that the presence of AFB_1_ was limited, showing small values. These results indicate that natural pastoralism was not the main cause behind AFM_1_ contamination of camel milk samples.

### 3.4. Aflatoxin Determination in Manufactured Feeds

The next step involved the investigation of manufactured dried feed material imported for use as camel feed. The manufactured dried feed materials consumed as camel feed were analyzed, and the results are shown in Table 5. AFB_1_ was present in the investigated samples; however, AFB_1_ was present in dry feed samples at high contamination levels. Changes in AFB_1_ determination using the two techniques of ELISA or VICAM were recorded as limited and fluctuated only slightly. These results may reveal that the consumption of manufactured feed was the source of the AFM_1_ contamination of camel milk samples.

### 3.5. Determination of Antioxidant Activity

**Figure 1:**
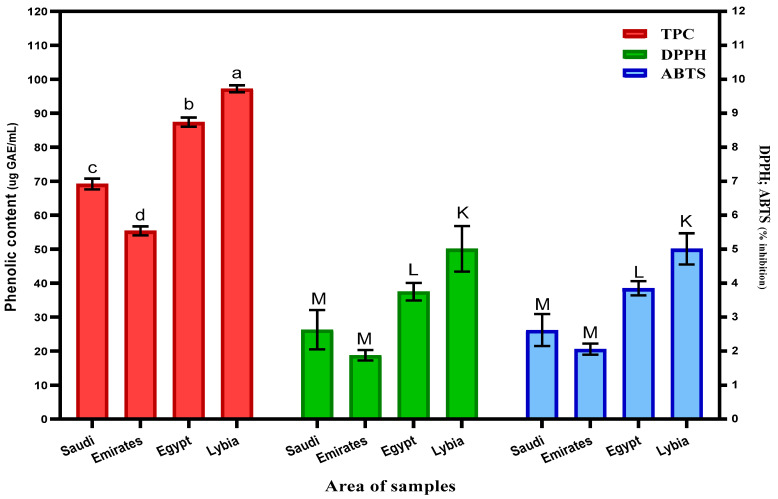
Total phenolic content and antioxidant activity for the collected camel milk from the Arabian Peninsula and North African regions. The columns with the same superscript letters show non-significant differences (*p =* 0.05). TPC: total phenolic compound contents determined as microgram Gallic acid equivalents per milliliter of milk sample. DPPH: DPPH (2, 2-diphenyl-1-picryl-hydrazyl-hydrate free radical solution), ABTS+: ABTS+ scavenging (2, 2′-Azinobis [3-ethylbenzothiazoline-6-sulfonic acid]-diammonium salt).

The total phenolic content and antioxidant activity of camel milk were part of our bioactivity investigation of the camel milk. The collected samples of camel milk were investigated for their antioxidant activity using two assays (DPPH and ABTS^+^). Furthermore, the total phenolic content of camel milk samples was determined to reflect their partial activity as antioxidants. The results (Figure 1) showed that camel milk samples collected from North Africa were distinct in their total phenols and antioxidant activity content. Additionally, the samples collected from Libya for contained more antioxidants than those collected from Egypt.

The samples collected from the Arabian Peninsula were lower in their levels of antioxidants compared to North Africa. Camel milk samples from the Arabian Peninsula were collected from the Kingdom of Saudi Arabia and the United Arab Emirates. The low content of antioxidants in these samples may be due to the consumption of these components to maintain the product’s quality and safety against microbial contamination during production or handling; it is not caused by any inherent lack of essential elements in the camel milk of these regions. The antioxidant activity of the food product is known to play a vital function in delaying microbial spoilage. Again, the primary type of feeding, such as using wild plants, might contribute to these results due to their bioactive components.

### 3.6. Antifungal Activity of Applied Probiotic Strains

**Figure 2:**
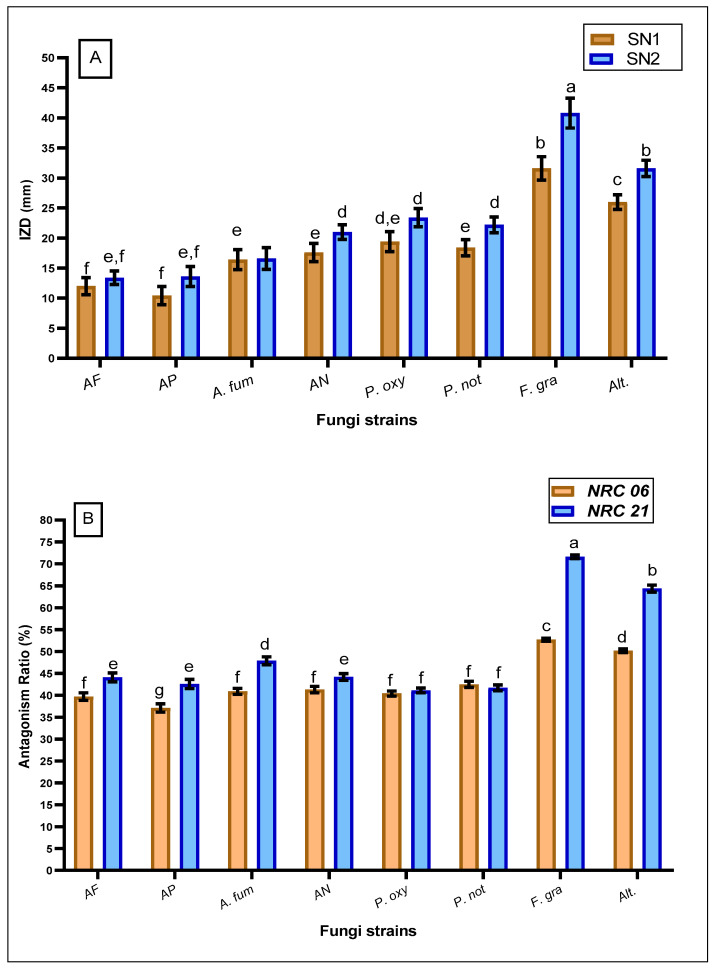
Antifungal activity of applied probiotic strains against toxigenic fungal strains. (**A**) Antifungal activity evaluated with bacterial supernatants determined according to zone inhibition diameter. (**B**) Antifungal activity of bacterial cells determined according to antagonistic ratio (%). For each of the (**A**,**B**), the columns with different superscript letters show significantly differences. SN1: supernatant of *L. acidophilus* NRC06; SN2: supernatant of *L. plantarum* NRC21; NRC06: bacterial cells of *L. acidophilus* NRC06; NRC 21: bacterial cells of *L. plantarum* NRC21. AF: *Aspergillus flavus*; AP: *A. parasiticus*; A. fum: *A. fumigatus*; AN: *A. niger*; P. oxy: *Penicillium oxysporium*; P. not: *P*. *notatum*; F. gra: *Fusarium graminarum*; Alt: *Alternaria alternata*.

The antifungal activities of the two applied strains (*L. acidophilus* NRC06 and *L. plantarum* NRC21) were evaluated using two assays, and two representative methods expressed the obtained results. The supernatant collected from the bacterial growth was applied using a well-diffusion assay. The activity in this method is described as inhibition zone diameter, which is recorded in Figure 2A. The results show that the strains possessed high inhibition zone diameters, particularly for *Fusarium* and *Alternaria*: two toxigenic fungi strains under investigation. Other fungi growth was recorded as being inhibited by lower levels, but they were still significant compared to the control (complete fungal growth).

Bacterial cells used antagonistically to stop the growth of toxic fungi were successful according to our results (Figure 2B). For the two strains, the effect of bacterial cells as inhibitors of *Aspergillus* and *Penicillium* fungi ranged from 40 to 50%. This ratio, however, has been documented to be up to 70% or more for some fungi, such as for the genus *Fusarium*. It was noticed that the inhibition influence was efficiently detected by utilizing two bacterial strains against eight strains of toxigenic fungi.

### 3.7. Anti-Aflatoxigenic Effects of Bacterial Strains

The results in Table 6 show the extent to which the strain of fungus (*A. parasiticus ITEM 11*), which is known to highly produce aflatoxins, was affected by the presence of probiotic bacteria in the fungal growth media. The effect on the fungus strain, associated with the presence of bacteria, was shown to exhibit a reduction in fungi mycelial growth and in aflatoxin secretion levels in the growth media compared to the control growth media.

The data reflected a high ratio of mycelial growth reduction at 41.003% ± 0.013 using the bacterial strain NRC 06. This inhibition ratio increased to 52.83% ± 0.07 by applying the NRC 21 bacterial strain. The reduction in aflatoxin concentration in the fungal growth media ranged between 84.39% ± 2.59 and 90.4% ± 1.32 for the utilization of bacterial treatment.

### 3.8. Aflatoxin Reduction in Spiked Camel Milk Inoculated by Bacteria

The camel milk samples collected from Egypt were chosen as median samples for the present part of the study. The samples utilized for the subsequent steps were collected from pastoral nomads of the northwestern desert area (Matruh to Siwa).

The previous strains of bacteria, which were recorded to have antifungal and anti-aflatoxigenic impacts, were tested in spiked samples of camel milk. Table 7 shows the applied strains’ capability to remove the aflatoxin content from camel milk. Moreover, the aflatoxin removal results from samples indicated that the approach efficiency is high. Aflatoxin removal using bacterial strains was recorded for aflatoxin B_1_ and aflatoxin M_1_. The efficiency of the NRC 21 bacterial strain for Aflatoxin removal was 100% as the treated sample recorded detected no Aflatoxins. The results reveal that there was more bacterial efficiency in removing toxins from individual spiked samples than in the mixed spiked samples. However, the gap between removing toxins from individual samples or mixed-toxin samples was still limited.

## 4. Discussion

Camel milk is one source of biologically active substances because it may contain functional proteins and peptides that have activity against several pathogens [22]. Camel milk and its production areas are often associated with Bedouins and desert regions, as it is known for its widespread use in those areas [23]. Camel milk peptides are linked to nutraceutical impacts when consumed [21,24]. Camel milk could be contaminated by food hazards, like other milk types, and these contaminants may turn it from a source of health benefits to a source of health issues. Camel milk could be contaminated due to production, handling, transportation, storage, or marketing conditions. While good hygiene practices are required for safe production, this may be difficult to apply in some production areas, which affects the safety of camel milk.

Aflatoxins are a significant hazard, considering their classification as pre-carcinogens [25,26]. These chemical hazards may contaminate camel milk directly (AFM secreted from AFB biotransformation) or indirectly through cross-contamination with AFB toxins [27]. Collected samples of camel milk were investigated for both contamination types (direct or indirect), and the results showed evidence of both types (Table 1 and Table 2). Two geographical areas were included in the present study: the Arabian Peninsula and North Africa. Two countries represented each area for the collected samples along with six regions (three regions for each country). The results reflected complete contamination of the collected camel milk samples (with AFB_1_ and AFM_1_). The presence of aflatoxins in milk samples in ascending order according to concentration was as follows: Libya < Egypt < Saudi Arabia < United Arab Emirates. This result shows that the Arabian Peninsula was a more hazardous production area compared to North Africa. The aflatoxin concentrations in tested samples were evaluated using a VICAM fluorometer and ELISA reader. The method validation of each approach was evaluated to ensure result accuracy (Table 3). Feed materials were investigated, including wild plant feed and manufactured feed imported from outside the country.

Aflatoxin contamination in camel milk has been previously tested in camel milk samples by other researchers [28,29,30]. Still, these studies are few in number, and none of them studied the relationship between the contamination of feed in the places of production and the levels of aflatoxins in the milk produced in the same areas.

The current study evaluated the two types of feed used for camels. The results indicated high levels of aflatoxin B_1_ contamination in the manufactured feed abundant in the Arabian Gulf region. Additionally, manufactured fodder is the alternative source in case of scarcity of wild plants and during drought periods throughout the year. The results highlight the high level of AFB_1_ detected in the manufactured feed type (Table 5). From these results, it could be concluded that the primary source of hazard for aflatoxin contamination is manufactured feed. The low level of aflatoxin contamination in wild plant feed may be linked to their bioactive components. These components play defensive roles on behalf of the plants and preserve their spoilage. 

It is essential to search for a method that aids in removing aflatoxin contamination from camel milk and maintains its nutrition and health benefits. Several strategies have been applied to detoxify aflatoxin in dairy products, such as using non-traditional oils [31]. Probiotic strains could play this function and support the milk’s beneficial properties. Two isolates of local strains, *L. acidophilus* NRC 06 and *L. plantarum* NRC 21, were investigated to evaluate their antifungal activity. The strains showed a high inhibition impact against eight strains of toxigenic fungi (Figure 2). The application of bacterial supernatant using an agar-well diffusion assay showed an inhibition zone diameter range between 11–40 mm (Figure 2A). Using the bacterial cell antagonistic impact, the ratio of inhibition shown reached up to 70% (Figure 2B).

A bacterial genus of *Lactobacilli* previously known for presenting probiotic properties has been known to bind into pathogens and limit their growth [32,33]. Lactobacillus strains also produce several secondary metabolites, including bacteriocins, active peptides, hydrogen peroxide, and organic acids. Bacteriocins of *lactobacilli* strains, such as *L. plantarum*, have been previously characterized. Pure substances were tested for their antifungal and anti-aflatoxigenic impacts [34,35]. The bioactivity characteristics of *L. plantarum* make it a distinguishable application for food preservation [36]. Incorporating probiotic bacteria as an antifungal agent in food may minimize the incidence of fungal spoilage and toxicity, and it may also extend shelf life and reduce mycotoxin concentrations [37]. The presence of probiotics in food might change its physiochemical and organoleptic features. These changes may be linked to the impacts referred to previously. In contrast, the predominant population of fungi infecting a typical meal should be considered when choosing the most effective probiotics/combinations of probiotic bacteria to prevent fungal development [7,10]. The reason for this is that antifungal activities of probiotics are fungal-strain-specific, which means that a probiotic strain may be very active against one fungus strain while having no impact on the growth of another [32,36].

Previous results have shown that strain differences in AFB_1_ removal are unequal as bacterial strains are differentiated in their activity [6]. Contrary to Gram-negative bacteria, Gram-positive bacteria removed aflatoxin more efficiently [6,38]. It is also worth noting that a study conducted by Line and Brackett [39] pointed out that the concentration and the growth stage of the cells applied, besides the incubation time, possessed a function in the elimination rates of mycotoxins as well as in the efficiency of their removal from the growth media.

It is clearly shown from this study that both NRC 06 and NRC 21 have significant effects in inhibiting toxigenic fungal contamination in growth media (Table 6). Additionally, these strains could reduce aflatoxin levels of AFB_1_ and AFM_1_ in liquid media (Table 6) and in spiked samples of camel milk (Table 7). Both NRC 06 and NRC 21 are classified as probiotic strains. Although these strains were recorded to detoxify aflatoxins, they can also remove other mycotoxins. The present bacterial strains are a potential approach for reducing aflatoxin during the food pathway metabolism in the gastrointestinal tract. The application of investigated bacteria to remove aflatoxin as in individual or in combination contexts (AFB_1_ + AFM_1_) in spiked samples of camel milk could recommend their utilization as a fast treatment for camel milk before consumption. This study also recommends the fermented consumption of camel milk instead of fresh consumption due to the high contamination recorded in the collected samples.

## 5. Conclusions

Camel milk is a beneficial dairy product consumed widely for its nutritional and health benefits. Recently, aflatoxin contamination has been known to threaten several food products, including dairy food materials. Camel milk samples were collected from the Arabian Peninsula and North Africa and contaminated. Samples analyses using two validated techniques (ELISA and Fluorometer) indicated the presence of AFB_1_ and AFM_1_. The AFM_1_ in camel milk was high in the Arabian Peninsula region. Cross-contamination with AFB_1_ was also recorded. However, feed material was recorded as positively contaminated. Two probiotic strains of NRC 06 and NRC 21 showed distinguished antifungal activity. These strains were able to inhibit the growth of eight toxigenic fungi strains. They also removed aflatoxin from the simulated media. Finally, the NRC 06 and NRC 21 bacterial strains effectively reduced aflatoxin content whether applied individually or in mixtures to spiked camel milk after incubation treatment. Based on these results, we recommend the fermentation of camel milk using probiotic strains as an approach to limit aflatoxin contamination in camel milk. Further studies are also recommended to find suitable solutions to aflatoxin contamination in dairy products.

## Figures and Tables

**Table 1:** Determination of aflatoxin M_1_ in camel milk collected from two regions, the Arabian Peninsula and North Africa, evaluated using ELISA and VICAM techniques.

	AFM_1_ Detected via ELISA (ng/L)			AFM_1_ Detected via VICAM (ng/L)		
	Region 1	Region 2	Region 3	Region 1	Region 2	Region 3
**Saudi**	205.8 ± 69.93 ^b^	166.6 ± 23.56 ^a^	150.8 ± 31.23 ^a^	206.6 ± 26.44 ^a^	168.2 ± 22.52 ^b^	152.8 ± 33.56 ^b^
**Emirates**	291.1 ± 73.13 ^a^	225.8 ± 65.53 ^a^	256.1 ± 40.4 ^a^	293.2 ± 74.86 ^a^	223.6 ± 39.84 ^a^	256.4 ± 62.24 ^a^
**Egypt**	312.2 ± 21.45 ^a^	177.4 ± 16.31 ^b^	124.4 ± 25.43 ^c^	314.8 ± 22.84 ^a^	178.0 ± 16.81 ^b^	124.6 ± 27.87 ^c^
**Libya**	124.4 ± 15.63 ^a,b^	66.2 ± 19.42 ^b^	99.4 ± 17.46 ^a,c^	128.8 ± 16.31 ^a,b^	70.6 ± 18.61 ^b^	100.0 ± 20.86 ^a,c^

The data were expressed as means ± SD (*n* = 5; *p* < 0.05). For each technique, the data with the same superscript letter in the same rows show no significant differences.

**Table 2:** Determination of aflatoxin B_1_ in camel milk collected from two areas, the Arabian Peninsula and North Africa, evaluated via ELISA and VICAM techniques.

	AFB_1_ Detected via *ELISA* (ng/L)			AFB_1_ Detected via *VICAM* (ng/L)		
	Region 1	Region 2	Region 3	Region 1	Region 2	Region 3
**Saudi**	80.8 ± 12.07 ^a^	112.6 ± 8.45 ^b^	108.0 ± 13.46 ^c^	82.2 ± 13.67 ^a^	113.2 ± 9.88 ^b^	108.1 ± 14.11 ^c^
**Emirates**	152.8 ± 54.28 ^a^	180.2 ± 18.56 ^a^	75.4 ± 13.69 ^b^	156.4 ± 54.55 ^a^	180.6 ± 38.35 ^a^	76.0 ± 13.56 ^b^
**Egypt**	57.8 ± 8.24 ^b^	101.6 ± 13.33 ^a,b^	108.8 ± 21.66 ^a^	58.8 ± 6.79 ^a^	101.2 ± 13.91 ^b^	106.0 ± 21.56 ^b^
**Libya**	61.2 ± 15.52 ^a,b^	80.8 ± 6.7 ^a^	42.4 ± 8.86 ^b^	64.6 ± 16.9 ^a,b^	80.4 ± 5.57 ^a^	41.8 ± 8.69 ^b^

The data were expressed as means ± SD (*n* = 5; *p* < 0.05). For each technique, the data with the same superscript letter in the same rows show no significant differences.

**Table 3:** Data validation for the samples using VICAM and ELISA techniques to determine AFM_1_ and AFB_1_ recovery.

Spiked	ELISA Technique		Coefficient Variation (%)	VICAM Technique		Coefficient Variation (%)	Samples (*n*)
	AF Determined (pg/L)	AF Recovered (%)		AF Determined (pg/L)	AF Recovered (%)		
**AFM_1_**							
**10**	10.01 ± 0.01	100 ± 0.01	0	10.01 ± 0.02	100 ± 0.02	0	7
**20**	19.98 ± 0.03	99.9 ± 0.03	0.1	19.96 ± 0.02	99.8 ± 0.03	0.2	7
**40**	39.87 ± 0.05	99.67 ± 0.02	0.33	39.89 ± 0.08	99.73 ± 0.02	0.27	7
**80**	79.74 ± 0.11	99.67 ± 0.05	0.33	79.79 ± 0.14	99.74 ± 0.11	0.26	7
**160**	159.52 ± 0.28	99.7 ± 0.14	0.3	159.64 ± 0.11	99.78 ± 0.14	0.22	7
**AFB_1_**							
**10**	10.01 ± 0.01	100 ± 0.01	0	10.01 ± 0.01	100 ± 0.01	0	7
**20**	19.99 ± 0.01	99.95 ± 0.02	0.05	19.99 ± 0.02	99.95 ± 0.02	0.05	7
**40**	39.89 ± 0.12	99.73 ± 0.03	0.27	39.93 ± 0.06	99.83 ± 0.09	0.17	7
**80**	79.65 ± 0.23	99.56 ± 0.18	0.44	79.77 ± 0.1	99.71 ± 0.22	0.29	7
**160**	159.21 ± 0.47	99.5 ± 0.21	0.5	159.54 ± 0.34	99.71 ± 0.16	0.29	7

The data were expressed as means ± SD (*n* = 7; *p* < 0.05). AF: aflatoxin; AFM_1_: aflatoxin M_1_; AFB_1_: aflatoxin B_1_.

**Table 4:** Determination of Aflatoxin B_1_ in plant feed materials collected from camel pasture areas of the Arabian Peninsula and North Africa.

	AFB_1_ Detected via *ELISA* (ng/kg)			AFB_1_ Detected via *VICAM* (ng/kg)		
	Region 1	Region 2	Region 3	Region 1	Region 2	Region 3
**Saudi**	5.2 + 1.18 ^a^	9.4 + 1.71 ^b^	7.2 + 1.74 ^a,b^	10.2 + 2.49 ^a^	6.4 + 1.95 ^b^	9.6 + 2.36 ^a^
**Emirates**	12.2 + 3.05 ^a^	5.2 + 2.55 ^c^	7.8 + 1.80 ^b^	13.8 + 2.59 ^a^	6.2 + 6.95 ^b^	5.2 + 1.77 ^b^
**Egypt**	8.8 + 3.28 ^a^	8.0 + 3.76 ^a^	8.0 + 2.06 ^a^	10.8 + 1.48 ^a^	10.4 + 3.51 ^a^	9.4 + 2.83 ^a^
**Libya**	5.0 + 3.10 ^a^	4.0 + 2.05 ^a^	6.6 + 2.07 ^a^	7.8 + 0.84 ^a^	6.0 + 1.81 ^a^	8.0 + 2.34 ^a^

The data were expressed as means ± SD (*n* = 5; *p* < 0.05). For each technique, the data with the same superscript letter in the same rows show no significant differences.

**Table 5:** Determination of Aflatoxin B_1_ in manufactured feed collected from camel pasture areas of the Arabian Peninsula and North Africa.

	AFB_1_ Detected via *ELISA* (ng/kg)			AFB_1_ Detected via *VICAM* (ng/kg)		
	Region 1	Region 2	Region 3	Region 1	Region 2	Region 3
**Saudi**	376.6 ± 73.96 ^a^	461.2 ± 75.49 ^a^	377.6 ± 48.86 ^a^	378.2 + 73.19 ^a^	463.0 + 72.42 ^a^	381.0 + 109.08 ^a^
**Emirates**	732.4 ± 159.51 ^a^	646.2 ± 81.38 ^a^	719.0 ± 165.6 ^a^	731.2 + 157.28 ^a^	645.2 + 81.10 ^a^	719.2 + 161.27 ^a^
**Egypt**	437.8 ± 49.70 ^a^	360.4 ± 70.44 ^a^	416.2 ± 93.03 ^a^	438.0 + 50.19 ^a^	363.6 + 69.79 ^a^	417.6 + 93.07 ^a^
**Libya**	365.6 ± 64.22 ^a^	367.6 ± 58.04 ^a^	321.4 ± 68.83 ^a^	365.0 + 67.26 ^a^	368.0 + 59.01 ^a^	321.2 + 69.81 ^a^

The data were expressed as means ± SD (*n* = 5; *p* < 0.05). For each technique, the data with the same superscript letter in the same rows show no significant differences.

**Table 6:** Anti-aflatoxigenic effects of bacterial strains against the fungal growth of *A. parasiticus* and toxin production reduction in growth media.

	Flasks Containing NRC 06—Strain	Flasks Containing NRC 21—Strain	Control Flasks
**Mycelia weight** (g)	3.1117 ± 0.144 ^b^	2.4876 ± 0.208 ^c^	5.2741 ± 0.131 ^a^
**MIR** (%)	41.003 ± 0.013 ^b^	52.83 ± 0.07 ^a^	--
**AFB_1_** (ng/mL)	76.11 ± 14.37 ^b^	46.77 ± 9.81 ^c^	487.6 ± 12.48 ^a^
**RR—AFB_1_** (%)	84.39 ± 2.59 ^b^	90.40 ± 1.32 ^a^	--

The data were expressed as means ± SD (*n* = 5; *p* < 0.05). The data with the same superscript letter in the same rows show no significant differences. NRC06: bacterial cells of *L. acidophilus* NRC06; NRC 21: bacterial cells of *L. plantarum* NRC21. MIR: mycelial inhibition reduction; RR—AFB_1_: reduction ratio recorded for aflatoxin B_1_ concentration.

**Table 7:** Aflatoxin reduction using spiked camel milk fermented using bacterial strains of NRC 06 and NRC 21.

	AFB_1_—Spike CM (ng/mL)	AFM_1_—Spiked CM (ng/mL)	CM Containing AF Mixture	
			AFB_1_ (ng/mL)	AFM_1_ (ng/mL)
**Spiked control**	482.8 ± 5.24 ^a^	299.2 ± 6.13 ^a^	492.5 ± 3.71 ^a^	316.9 ± 2.14 ^a^
**NRC 06**	103.61 ± 7.64 ^b^	54.71 ± 6.84 ^b^	91.34 ± 4.55 ^b^	76.18 ± 6.24 ^b^
**NRC 21**	ND	ND	23.66 ± 4.89 ^c^	5.94 ±3.17 ^c^

The data were expressed as means ± SD (*n* = 5; *p* < 0.05). The data with the same superscript letter in the same columns show no significant differences. For each column, the result with different superscript letters were significantly different.

## Data Availability

The data used to support the findings of this study are included in the article.
